# Tracking vegetation phenology across diverse North American biomes using PhenoCam imagery

**DOI:** 10.1038/sdata.2018.28

**Published:** 2018-03-13

**Authors:** Andrew D. Richardson, Koen Hufkens, Tom Milliman, Donald M. Aubrecht, Min Chen, Josh M. Gray, Miriam R. Johnston, Trevor F. Keenan, Stephen T. Klosterman, Margaret Kosmala, Eli K. Melaas, Mark A. Friedl, Steve Frolking

**Affiliations:** 1 Harvard University, Department of Organismic and Evolutionary Biology, Cambridge, MA 02138, USA; 2 Northern Arizona University, School of Informatics, Computing and Cyber Systems, Flagstaff, AZ 86011, USA; 3 Northern Arizona University, Center for Ecosystem Science and Society, Flagstaff, AZ 86011, USA; 4 University of New Hampshire, Earth Systems Research Center, Durham, NH 03824, USA; 5 Boston University, Department of Earth and Environment, Boston, MA 02215, USA; 6 North Carolina State University, Department of Forestry and Environmental Resources, Raleigh, NC 27695, USA; 7 Lawrence Berkeley National Laboratory, Earth and Environmental Sciences, Berkeley, CA 94720, USA

**Keywords:** Ecosystem ecology, Phenology

## Abstract

Vegetation phenology controls the seasonality of many ecosystem processes, as well as numerous biosphere-atmosphere feedbacks. Phenology is also highly sensitive to climate change and variability. Here we present a series of datasets, together consisting of almost 750 years of observations, characterizing vegetation phenology in diverse ecosystems across North America. Our data are derived from conventional, visible-wavelength, automated digital camera imagery collected through the PhenoCam network. For each archived image, we extracted RGB (red, green, blue) colour channel information, with means and other statistics calculated across a region-of-interest (ROI) delineating a specific vegetation type. From the high-frequency (typically, 30 min) imagery, we derived time series characterizing vegetation colour, including “canopy greenness”, processed to 1- and 3-day intervals. For ecosystems with one or more annual cycles of vegetation activity, we provide estimates, with uncertainties, for the start of the “greenness rising” and end of the “greenness falling” stages. The database can be used for phenological model validation and development, evaluation of satellite remote sensing data products, benchmarking earth system models, and studies of climate change impacts on terrestrial ecosystems.

## Background & Summary

Vegetation phenology—the seasonal progression of plant activity through stages of dormancy, active growth, senescence, and back to dormancy—is a key regulator of both ecosystem processes and biosphere feedbacks to the climate system^1,2^. Phenology has been shown to be a highly sensitive indicator of the biological impacts of climate change on terrestrial ecosystems^3^. For example, in many summer-active temperate and boreal ecosystems, earlier spring onset and delayed autumn senescence have been observed in recent decades^4,5^. By extending the length of the growing season, these shifts in phenology also have important implications for ecosystem productivity and carbon cycling^6,7^. However, existing phenological models are poor^8^, leading to substantial uncertainties in projections of phenological responses to future climate change^9^.

The traditional method of collecting data on plant phenology has been for a human observer to monitor individual organisms on a regular interval (commonly once or twice per week) and record visually apparent changes in phenological state, such as budburst or flowering^10,11^. In the 1970s, the development of satellite remote sensing opened up new opportunities for global monitoring of phenology at the landscape scale^12,13^. Near-surface remote sensing, using radiometric instruments or imaging sensors mounted in close proximity to the land surface, complements these methods, at an intermediate (canopy-level) scale^14^.

**Figure 1:**
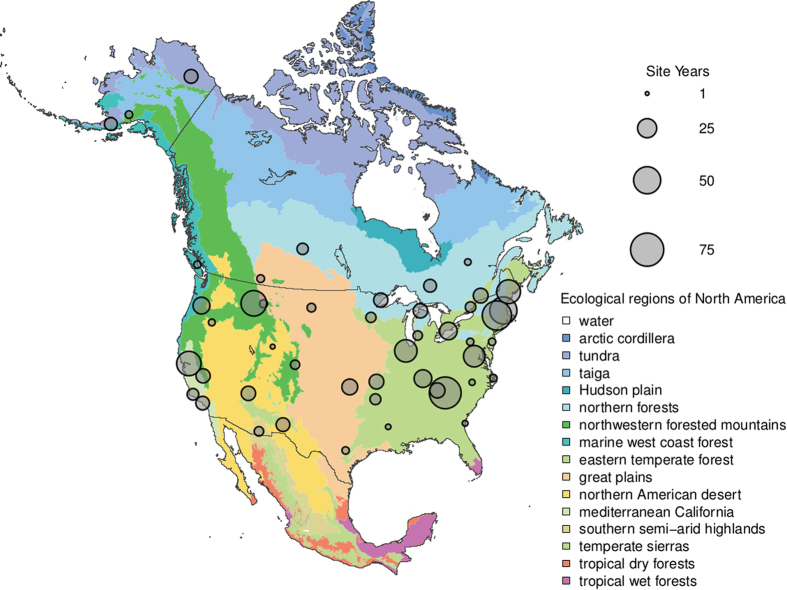
Spatial distribution of PhenoCam data across ecological regions of North America. Background map illustrates USA Environmental Protection Agency Level I Ecoregions^35^. Data counts have been aggregated to a spatial resolution of 4°, and the size of each circle corresponds to the number of years of data. Sites in Hawaii (6 site years), Panama (5 site years), and Europe (15 site years) are not shown.

Methods to extract phenological information from repeat photographs recorded with very simple imaging sensors—conventional, visible-wavelength, digital cameras—have been developed within the last decade^15^, and have been widely adopted^14,16^. Initial proof-of-concept work in a temperate deciduous forest demonstrated the viability of tracking deciduous forest phenology by calculating a canopy greenness index (the green chromatic coordinate, *G*_cc_)^15^ from the red, green and blue (RGB) pixel values in an image time series^17^. In a boreal forest dominated by evergreen conifers, subsequent measurements demonstrated that, over seasonal time scales, there was a surprisingly strong correlation between subtle shifts in the colour of the evergreen canopy and canopy-level photosynthesis estimated from eddy covariance CO_2_ flux meaurements^18^. These studies motivated the development of the PhenoCam network, which was established in 2008 to provide automated monitoring of vegetation phenology in forested ecosystems of the Northeastern United States and adjacent Canada. Since then, the scope and mission of the network has expanded to serve as a long-term, continental-scale, phenological observatory. Imagery from over 400 cameras deployed across North America, spanning a wide range of ecoregions, climate zones, and plant functional types (Fig. 1), is being uploaded to the PhenoCam server at least once daily (and in some cases as frequently as every 15 min), with imagery and derived data products displayed in near-real time on the project web page (http://phenocam.sr.unh.edu/). The archived images (at present, ca. 15 million images, requiring 6 Tb of disk space) provide a permanent record that can be visually inspected to determine the phenological state of the vegetation at any point in time. Quantitative data on the colour of vegetation—a proxy for its phenological state—can also be extracted from the images using simple image processing methods.

The data sets presented here (Data Citation 1) are derived from the analysis of imagery (Data Citation 2) from over 130 cameras, together totalling almost 750 years of data across a dozen different vegetation type classifications. In addition to automated quality control routines (e.g. filtering and outlier detection, described below), each time series has been visually evaluated and vetted for consistency and overall quality. For researchers interested in analysing the entire seasonal trajectory of canopy colour, which reflects both the amount of leaf area and the colour of individual leaves^19^, we include an “all-image” dataset, as well as more highly processed products in which data are aggregated to 1- and 3-day time steps using previously published methods^15^. Alternatively, for researchers interested in specific phenophase transition dates, we provide date estimates, with confidence intervals, for the start of the “greenness rising” and the end of the “greenness falling” stages^20^.

Data derived from PhenoCam imagery have been previously used to evaluate satellite phenology products^20–22^, to constrain and test new phenology models^23,24^, to understand relationships between canopy phenology and ecosystem processes^25–27^, and to study the seasonal changes in leaf-level physiology that are associated with changes in leaf color^19^. Given the lack of multi-year, standardized, and geographically distributed phenological data for North America^28^, we anticipate that these datasets will be widely used by researchers in plant ecology and plant physiology, community and ecosystem ecology, remote sensing and geography, and global change biology and Earth system science. Shifts in phenology are a particularly tangible example of the biological impacts of climate change, and thus these data may also find use in science education and outreach to the general public^29^.

## Methods

### The PhenoCam network

The PhenoCam network uses digital camera imagery to monitor ecosystem dynamics over time. Most of the cameras in the network are deployed within North America, from Alaska to Texas, and from Maine to Hawaii (Fig. 1), although a small number of cameras are located on other continents. Among vegetation types, deciduous broadleaf forests (392 site-years of data in the dataset), grasslands (121 site-years), and evergreen needleleaf forests (80 site-years) are the best represented, with other vegetation types being less well represented (Table 1). However, the network continues to grow as new cameras are added, and increasing network coverage in under-represented ecosystems is a priority.

All data presented here have been derived from archived PhenoCam imagery. The images themselves are available for online viewing or download through the PhenoCam project web page (e.g., http://phenocam.sr.unh.edu/webcam/browse/<sitename>), and the methods summarized here have been previously described in the literature^14,15,19^. The Data Records described here have been prepared using imagery through 31 December 2015, and we note that this imagery has also been submitted for permanent and secure archiving (Data Citation 2). However, most of the sites continue to be active (see Data Record 1), and new sites are being added to the network on a regular basis. Thus, we anticipate releasing periodic updates to this dataset in coming years.

Sites included here have been judged to have appropriately high-quality and reliable imagery such that the seasonal patterns of vegetation phenology are sufficiently well characterized to merit inclusion here. This assessment has been based on both visual inspection and quantitative analysis of the image time series, and resulting data products, by the authors of this Data Descriptor.

Within the PhenoCam network we distinguish three site classes, which we refer to here as Type I, Type II and Type III sites. Briefly, Type I sites follow a standard protocol and site personnel are directly engaged in the deployment and maintenance of the camera, whereas at Type II and Type III sites, either one or both of these criteria is waived. Basic information about each PhenoCam site (site class, location, start and end dates of site imagery, camera model, vegetation type, climate, and other measurements) is contained in Data Record 1 (see below).

Type I sites use a prescribed camera (NetCam SC IR, StarDot Technologies, Buena Park, CA, USA), configured (https://khufkens.github.io/phenocam-installation-tool/) and deployed (https://phenocam.sr.unh.edu/pdf/PhenoCam_Install_Instructions.pdf) according to a standard protocol. Critical aspects of this protocol are as follows. First, and most importantly, cameras are set to fixed white balance. Second, cameras are mounted to a secure point (typically a tower, mast, or building) that is taller than the vegetation of interest, so that the camera’s field of view is across the landscape, and cameras are inclined downward at 20°–40°. Ideally, the vegetation of interest dominates the image, but typically some sky is included. Third, cameras are pointed north (in the northern hemisphere) to minimize lens flare, shadows, and forward scattering off the canopy. Commonly, there are additional measurements (ground observations of phenology, leaf area index, CO_2_/H_2_O fluxes, etc.) that are also made onsite; these are documented in the site metadata that comprise Data Record 1. Importantly, at Type I sites, site representatives are actively involved in maintaining the camera and ensuring data quality and continuity.

The primary difference between Type I and Type II sites is that Type II sites do not use the standard NetCam SC IR camera. However, at Type II sites, other elements of the deployment protocol are followed as closely as possible. In particular, we emphasize the importance of setting the camera to fixed white balance, which on some cameras is referred to as “fixed” or “manual" colour balance, or “daylight” mode. And, as at Type I sites, site personnel are actively involved in camera maintenance and are engaged to ensure data continuity and quality.

At Type III sites, cameras have already been deployed (typically by federal or state agencies, research organizations, and in some cases by individuals or businesses), and the imagery has been made publicly available online. For these sites, we have judged the field of view to be relevant for current PhenoCam objectives, and the imagery to be of sufficiently high quality to be worth including in the PhenoCam archive (note that more inclusive online webcam archives exist, e.g. AMOS, the Archive of Many Outdoor Scenes^30^), but the standard protocol is not followed explicitly, and we have limited contact with site personnel. However, where possible, we worked to obtain the full (historical) record from these cameras (to 2002 or earlier, in some cases).

For all classes of cameras, high-frequency (typically every 30 min, although in some cases more or less frequently, e.g. daily) camera imagery is stored on the PhenoCam server as a 24-bit (8 bits per channel) JPEG image. Image dimensions range from 640×480 pixels (0.3 megapixels, e.g. site “bartlett”) to 4000×2500 pixels (10.0 megapixels, e.g. site “coville”).For Type I sites, images are 1296×960 pixels (1.3 megapixels). JPEG compression is minimal for Type I sites. The site name, as well as a date and time stamp (in local standard time), is embedded in the filename of every image, and since 2014 all new Type I cameras have been programmed to upload a metadata file simultaneously with every image file. Every night, any new images that have been uploaded to the server during the previous 24 hours are copied to the data archive, and then processed and analysed as described below. The processing is conducted using scripts coded in Python. Scripts used for image processing, including extraction of colour information, and generation of “all-image” (Data Record 3) and “summary product”(Data Record 4) time series data files, are available at https://github.com/tmilliman/python-vegindex/ with an open source license agreement.

### Image analysis and data processing

Image analysis consists of several steps. First, an appropriate “region of interest” (ROI) is defined, corresponding to the area within each digital image for which colour information will be extracted. Here, we have selected ROIs to characterize the dominant vegetation type in each image. For sites where more than one vegetation type could be clearly identified, we also selected secondary ROIs. The ROI coordinate definitions are stored, in TIFF format, as a series of binary image masks, which comprise an ROI’s “mask sequence”. For each ROI mask sequence at each site, an “ROI list file” detailing the date and time range over which each mask is to be applied is contained in Data Record 2 (see below).

We used changes in the position of the horizon line of each image to diagnose camera field of view shifts. To facilitate this, we generated a composite image in which changes in the position of the horizon could be easily detected. Briefly, the composite image was produced by assigning the middle column of pixels from the mid-day image on day *i* to the *i*th column in the composite image. A separate composite image was produced for each calendar year of imagery for a site. We created a new mask to account for each field of view shift, with the goal of keeping vegetation within the ROI as consistent as possible. When there was a large field of view shift, a change in the camera model or camera settings, or any other exogenous event that resulted in a significant discontinuity in the extracted greenness time series, we created a new ROI list with a separate identification number tag. We have made every effort to verify that the derived time series are internally consistent, without obvious artefacts (e.g., baseline drift, step changes, and so forth) that would adversely affect the integrity of the time series.

The digital cameras used here all record JPEG images with colour information stored in three separate layers (red, green, and blue; RGB). According to the standard additive colour model, representation of any given colour in the visible range is achieved by varying the intensity (pixel value) of these primary colours. Thus, each pixel in the image is associated with a digital number (“DN”) triplet, with each element in the triplet corresponding to the intensity of one of the colour layers. Therefore, the second step in the image analysis was to read in the images, and associated mask sequence, and to characterize the frequency distribution of the RGB DN triplets within the ROI. We did this separately for each ROI at each site, to produce the “all-image” data files contained in Data Record 3 (see below).

For each image, the date and local time were extracted from the image file name. We also calculated the solar elevation angle based on the date and local time stamp, using standard formulas (Python package “pyephem”, http://rhodesmill.org/pyephem/).

We then characterized the frequency distribution of the RGB DN triplets within the ROI on a channel-by-channel basis. Thus, for each of the red, green and blue colour channels, we determined the mean and standard deviation, as well as the 5^th^, 10^th^, 25^th^, 50^th^ (median), 75^th^, 90^th^, and 95^th^ percentile values, of the DN distribution across all pixels in the ROI. We also calculated the pairwise correlation, across all pixels in the ROI, between red DN and green DN, red DN and blue DN, and green DN and blue DN, in order to provide basic information about the joint distributions of the three colour channels.

Overall, however, time series of RGB DN triplets are noisy, and of little use on their own for phenological analyses, because both external factors affecting scene illumination (weather and atmospheric effects, as well as solar illumination geometry) and internal processing (including exposure control) can confound the underlying seasonal signal^17,18^. This variability can be largely suppressed by converting the DN triplets to their respective chromatic coordinates (e.g. the green chromatic coordinate, *G*_cc_)^14,15^. Numerous studies have demonstrated the value of the *G*_cc_ index (and the corresponding red chromatic coordinate, *R*_cc_)^18^ for characterizing the seasonal trajectory of vegetation color and activity^14^. We therefore calculated *G*_CC_ as in Equation 1, where *X*_DN_ denotes the mean (across the ROI) digital number for colour channel *X*. We note that a variety of other vegetation indices can be calculated from the RGB DN triplets that are included in Data Record 3 (ref. 14).

(1)Gcc=GDNRDN+GDN+BDN

Over short time scales (i.e., a few days or less), the colour of vegetation tends to be relatively constant, and for many applications the high temporal frequency of the all-image data files is unnecessary. We therefore processed the all-image data files to 1- and 3-day “summary product” files, which are more readily used for analysis of seasonal patterns, and which are included in Data Record 4. Processing for both 1- and 3-day summary product files is identical, except that for the 1-day file, data are reported at a one-day time step, and statistics are calculated using only the valid images (i.e., passing the quality-control filters defined below) from that day. For the 3-day file, data are reported at a three-day time step, corresponding the middle day of the 3-day interval, but statistics are calculated using the valid images across the entire 3-day interval. The main advantage offered by the 1-day summary product is higher temporal resolution, whereas the data in the 3-day summary product tend to be less noisy because of a longer averaging period, and the greater likelihood of “optimal” illumination conditions for obtaining a good photograph of the canopy.

We report two sets of statistics in the summary product files contained in Data Record 4. The first set is calculated from the one image that was recorded closest to 12:00 noon, local standard time, on the reported day. The second set of statistics is calculated from all valid images within the 1- or 3-day interval that passed a series of quality-control filters: we excluded images obtained when the solar elevation was less than 10° above the horizon, images that were too bright (mean [red DN + green DN + blue DN] across the ROI > 665), and also images that were too dark (mean [red DN + green DN + blue DN] across the ROI < 100)^20,26^.

The mean and standard deviation (across all valid images) of each colour channel DN was calculated from the mean (across the ROI) values contained in the all-image file. Likewise, for *G*_cc_ and *R*_cc_, we calculated (across all valid images) the mean, standard deviation, and 50^th^, 75^th^ and 90^th^ percentile values of these two indices using the mean (across the ROI) values contained in the all-image file. Our rationale for including these percentile values, in addition to the mean, is that previous work^15^ has shown that under sub-optimal lighting conditions (associated with clouds, precipitation, and other adverse weather), the measured *G*_cc_ tends to be reduced compared to *G*_cc_ measured under sunny skies. As a result, the 90^th^ percentile *G*_cc_ value calculated across a 3-day moving window—which tends to track the mode of the data, without being overly influenced by outliers—was found to be substantially less noisy than other percentile values or mean mid-day values. However, in processing the data sets presented here, we have found that in some instances the 90^th^ percentile value tends to track the extreme upper values in the data too closely, meaning that it is more influenced by outliers than would be ideal. The 50^th^ and 75^th^ percentile values are thus presented—in addition to the mid-day and mean values—as an alternative for end-users of the data to consider. We recognize that the statistic that best characterizes the data may depend on not only the site in question but also the situation or science question at hand.

### Quality flags and outlier detection

Although the 90^th^ percentile *G*_cc_ value, as presented in the summary product files contained in Data Record 4, has been shown to be highly effective in reducing the impact of variation in weather, atmospheric effects, and illumination conditions^15^, some residual noise in the resulting time series is inevitable. We also acknowledge the presence of occasional large outliers, which can occur for a variety of reasons. Foremost among these is the presence of snow and ice, which is particularly problematic at sites dominated by evergreen vegetation: when green foliage is covered by white snow, the mean *G*_cc_ value across the ROI tends to be reduced, and sometimes substantially so. This is less of a problem at deciduous sites, because leafless branches tend towards grey in colour—and thus, like snow, have a *G*_cc_ value around 0.33.

Through an online crowdsourcing platform, Knowxel^31,32^, we engaged volunteers to visually inspect every midday image in the PhenoCam data archive (through the end of 2014), and to answer a simple question about the presence of snow in that image. For sites with trees, volunteers could choose one of four answers:

- There is **NOT snow** in the picture
- There is **snow COVERING** at least some of the trees
- There is **snow ONLY** on the ground
- **I CANNOT distinguish** whether there is snow or not (the image is blurry/foggy...)

This last category was included to identify images where the camera’s field of view was obscured for any reason. For sites without trees, volunteers chose from three answers:

- There is **NOT snow** in the picture
- There **IS snow** in the picture
- **I CANNOT distinguish** whether there is snow or not (the image is blurry/foggy...)

Three volunteers looked at each image and the majority answer was taken as the accepted classification. In 88% of the cases, the three volunteers were unanimous. For all images, 73% were classified as not having snow, 4% were treeless sites with snow, 9% had snow on the trees and the ground, 10% snow on the ground, but not on the trees, and 4% were obscured images. Of the 27% of images that had snow or were obscured images, 66% were classified unanimously. An expert team, selected from the co-authors of this paper, also evaluated a random representative subset of over 2,000 of these images (1% of the total), and in 97.0% of the cases, the expert team consensus agreed with the volunteer consensus. This accuracy increased to 98.4% when the location of the snow (on trees or on the ground) was ignored by combining the second and third categories for sites with trees. Here, we report the consensus (majority rule) volunteer evaluation for each midday image. We use the following codes: Not evaluated=NA; 1=bad or obscured image; 2=no snow in image; 3=snow (used for non-tree sites); 4=snow on ground only (used for treed sites); 5=snow on trees (and ground, used for treed sites). These can be used as quality flags to filter data points for which the extracted colour information may not be representative of the vegetation in the camera’s field of view.

Additionally, we ran an iterative, spline-based outlier detection algorithm on each of the *G*_cc_ time series (mean, 50^th^, 75^th^, and 90^th^ percentile values) included in the 1- and 3-day summary product files. First, using a range of smoothing factors, we fit a family of cubic smoothing splines to each of time series. We selected the optimal spline from within this family using Akaike’s Information Criterion (AIC), which balances goodness-of-fit against model complexity in order to avoid over-fitting. Specifically, we used a version of AIC that has been proposed for smoothing parameter selection in nonparametric regression^33^.Then, in an iterative process, we identified and flagged data points lying more than 4 standard deviations (calculated from the spline residuals, assuming a Laplace distribution) above, or 2 standard deviations below, the spline. We used this asymmetrical threshold because outliers below the spline tended to be larger in magnitude and more common than outliers above the spline, and we wanted to filter these more aggressively. With these outliers excluded, we then repeated the spline fitting process. This was done up to 20 times or until no further outliers were detected from one iteration to the next.

The spline fitting process was repeated one final time, so that together with the outlier flags, a smoothed and interpolated time series (with uncertainty estimates) could be used for transition date estimation (see below) and included in the files 1- and 3-day summary product files comprising Data Record 4. From this spline we also calculated the root mean squared error (RMSE) of the spline residuals, so that the signal-to-noise ratio (= seasonal amplitude/RMSE) could be used as a measure of data quality. RMSE values are reported in the header of the transition date files comprising Data Record 5.

### Transition date estimation

Using an approach similar to the “spline interpolation” method that has been previously applied to PhenoCam data^20^, we extracted phenophase transition dates for each ROI mask sequence. These are intended to define the start of the “greenness rising” and end of the “greenness falling” stage for afull cycle of vegetation activity (i.e., from dormancy, through green-up or “greenness rising”, peak activity, senescence or “greenness falling”, and back to dormancy). These dates are identified as changepoints using the Pruned Exact Linear Time (PELT) method^34^ for each cycle with a penalty factor (beta=0.5) and a minimum segment length of *n*=14 (days). Thus, unlike sigmoid-based approaches that have been typically used in the literature^12,20^, our method is not limited to ecosystems with a single annual cycle of green-up and senescence.

The AIC-selected smoothing spline, described in the previous section, is central to this method. The spline is first used to determine the *G*_CC_ minima (the baseline before a “greenness rising” stage or after a “greenness falling” stage) and maxima (the peak between “rising” and “falling” stages). From the *G*_CC_ minima and maxima associated with each stage, we then used the spline to identify the dates when 10%, 25%, and 50% of the amplitude (= maxima – minima) were reached. Transition date uncertainties were then derived from the confidence interval (1.96σ) around the smoothing spline, with a minimum uncertainty extending to the immediately preceding or following observation (i.e.,±1 and±3d for the 1- and 3-day *G*_CC_ time series, respectively; larger in the case of missing data on either side of the estimated transition date).

Our rationale for identifying these three different dates (rather than just a single date) is that while the 10% amplitude threshold might correspond most closely to the “true” onset of green-up, the most rapid rise (or fall) in greenness, and hence the most tightly-constrained transition date, tends to occur later (or earlier) during the rising (or falling) stage. By providing estimates of 10%, 25%, and 50% amplitude, we offer end-users of the data set the choice of the transition most appropriate for their specific application.

The “transition date” data files, derived from the 1- and 3-day summary product files of Data Record 4, are contained in Data Record 5 (see below). These include for each “greenness rising” or “greenness falling” stage both the transition dates and their uncertainties, as well as the associated *G*_CC_ minima, maxima, and threshold values (10%, 25% and 50% of amplitude). The *G*_CC_ minima and maxima can, for example, be used to normalize the data to a common 0-1 scale. This may facilitate cross-site comparisons of phenological patterns.

### Sample time series

**Figure 2:**
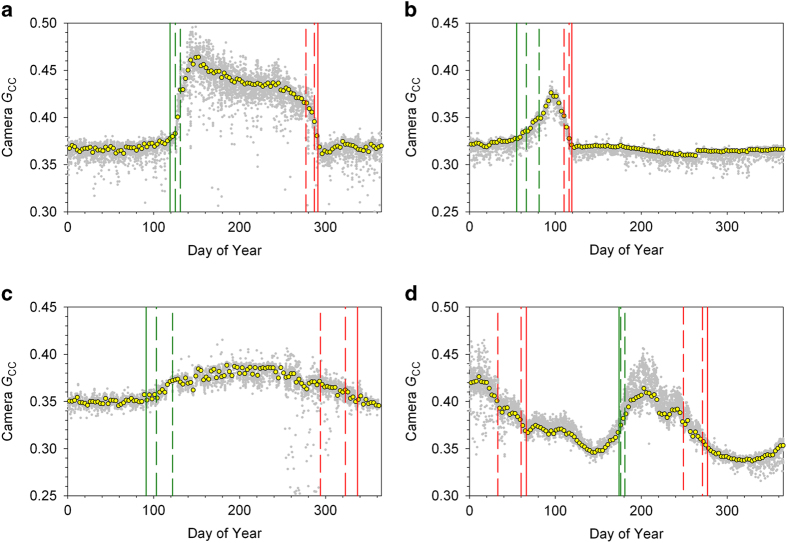
Example time series of camera *G*_CC_ (green chromatic coordinate) for different ecosystem types. Yellow circles are three-day median *G*_CC_ values; grey circles are all-image *G*_CC_ values; green lines denote phenological transition dates (10%, 25%, 50% amplitude) during “rising *G*_CC_” phase; red lines denote phenological transition dates (50%, 25%, 10% amplitude) during “falling *G*_CC_” phase. Sites: (**a**) site “harvard”: Harvard Forest, Massachusetts – temperate deciduous broadleaf forest; (**b**) site “jasperridge”: Jasper Ridge Biological Preserve, California – Mediterranean grassland; (**c**) site “howland1”: Howland Forest, Maine – temperate/boreal evergreen needleleaf forest; (**d**) site “kamuela”: Kamuela (Parker Ranch), Hawaii – tropical grassland.

Sample time series, for a variety of different ecosystem types, are shown in Fig. 2, where we illustrate (1) the all-image *G*_cc_ time series, which is then (2) processed to a 3-day summary product (here, the 50^th^ percentile value, or median, of the 3-day *G*_CC_), which is then (3) screened for outliers (for the time series shown here, no outliers were identified), before (4) identification of phenophase transition dates for the “greenness rising” and “greenness falling” stages of each cycle of vegetation activity.

## Data Records

The PhenoCam Dataset v1.0 consists of a set of 5 data records for each site–ROI (region of interest) combination. Described fully below, these are organized as follows for each PhenoCam site.

<sitename>

- └─── **data_record_1** (contains general metadata for each site)
- <sitename>_meta.json
- <sitename>_meta.txt
- └─── **data_record_2** (contains the ROI list files and image mask files used for image processing)
- <sitename>_<veg_type>_<ROI_ID_number>_roi.csv
- <sitename>_<veg_type>_<ROI_ID_number>_<mask_index>.tif
- └─── **data_record_3** (contains all-image time series of ROI colour statistics, calculated for every image in the archive, using data_record_2)
- <sitename>_<veg_type>_<ROI_ID_number>_roistats.csv
- └─── **data_record_4** (contains summary time series of ROI colour statistics, calculated for 1 and 3 day aggregation periods from data_record_3)
- <sitename>_<veg_type>_<ROI_ID_number>_1day.csv
- <sitename>_<veg_type>_<ROI_ID_number>_3day.csv
- └─── **data_record_5** (contains phenological transition dates, calculated from data_record_4)
- <sitename>_<veg_type>_<ROI_ID_number>_1day_transition_dates.csv
- <sitename>_<veg_type>_<ROI_ID_number>_3day_transition_dates.csv

Here, <sitename> is the name of each camera site, <veg_type> is a two-letter code defining the type of vegetation for which data have been processed, and <ROI_ID_number> is a unique identifier to distinguish between multiple ROIs of the same vegetation type for a given site. Together, these five data records are contained within Data Citation 1, and are derived from the imagery in Data Citation 2.

### Data record 1

Data Record 1 contains general metadata for the PhenoCam network sites from which processed imagery has been included here. To facilitate data access and re-use we include metadata files both in machine-readable JSON format and as standard text files, where fields are specified as key-value pairs. The naming convention for Data Record 1 files is as follows:

- <**sitename**>_meta.json
- <**sitename**>_meta.txt

The metadata fields are as follows:

- **last_updated**: the date (format: YYYY-MM-DD, where MM=01-12 and DD=01-31) on which the site metadata were last updated
- **project**: by default, all sites are associated with the PhenoCam Network project
- **project_url**: the URL of the PhenoCam project web page
- **fairuse_statement**: the general PhenoCam statement on data use, acknowledgment, and redistribution.
- **project_fairuse_url:** the URL of the PhenoCam fairuse_statement
- **sitename:** the name of the camera site, e.g. “coweeta,” used to designate all images and products associated with that site
- **long_name:** a more descriptive name for each camera site, e.g. “Coweeta Hydrologic Laboratory, USDA Forest Service, Southern Research Station, Otto, North Carolina”
- **lat:** the latitude (in decimal degrees) of the camera itself (*not* the centre of the camera’s field of view)
- **lon:** the longitude (in decimal degrees) of the camera itself (negative values indicate locations west of the prime meridian)
- **elevation:** the elevation of the ground surface (m above sea level) at the camera site
- **contact1, contact2**: the names and email addresses of the site representatives
- **active:** “True” if new images from the site are still being added (as of **last_updated** date of the metadata file) to the PhenoCam archive; “False” otherwise
- **date_start:** the date of the first image in the archive for this site (format: YYYY-MM-DD)
- **date_end:** the date of the most recent image (as of the **last_updated** date of the metadata file) in the archive for this site (format: YYYY-MM-DD)
- **nimage**: number of images in the archive for this site
- **site_type:** the site class (Type I, II, or III) as described previously in the Data Descriptor. Type I sites follow a standard protocol and site personnel are directly engaged in the deployment and maintenance of the camera
- **ir_enabled:** “Y” if the camera is capable of taking both visible and infrared (or visible+infrared) imagery (the visible+infrared images have not been processed for the current dataset release, but they are available for download from the PhenoCam project web page)
- **method**: sites for which images are pushed to the PhenoCam server via FTP are designated “ftppush”, those for which images are pulled from an external server are designated “httppull”
- **utc_offset:** the difference (in hours) between UTC (Coordinated Universal Time) and standard time at the site, e.g. −5 for sites on Eastern Standard Time.
- **camera_description:** the brand and model of the camera being used, e.g. StarDot NetCam SC for Type I sites
- **camera_orientation:** the compass direction in which the camera is pointing
- **group**: a number of camera sub-networks are designated, e.g. “LTAR” for those belonging to the LTAR (Long Term Agroecosystem Research) network
- **flux_data**: “True” if eddy covariance flux measurements are being (or have been) made at the site
- **flux_networks**: if the site belongs to a network (e.g., AmeriFlux, Fluxnet-Canada, etc.), then the network is identified
- **flux_sitenames:** FLUXNET site code, if applicable
- **ecoregion**: numeric code identifying the site’s EPA Ecoregion^35^ classification (source: https://www.epa.gov/eco-research/ecoregions-north-america), where: 0=Water; 1=Arctic Cordillera; 2=Tundra; 3=Taiga; 4=Hudson Plain; 5=Northern Forests; 6=Northwestern Forested Mountains; 7=Marine West Coast Forest; 8=Eastern Temperate Forests; 9=Great Plains; 10=North American Deserts; 11=Mediterranean California; 12=Southern Semi-Arid Highlands; 13=Temperate Sierras; 14=Tropical Dry Forests; 15=Tropical Wet Forest.
- **MAP_site, MAP_daymet, MAP_worldclim:** mean annual precipitation (mm) as reported by site personnel, and as from the Daymet^36,37^ (https://daymet.ornl.gov/) and WorldClim^38^(http://worldclim.org/) databases, respectively
- **MAT_site, MAT_daymet, MAT_worldclim:** as above, but for mean annual temperature (°C)
- **primary_veg_type:** the dominant vegetation type at the site (see Table 1 for abbreviations)
- **secondary_veg:** secondary vegetation type at the site (if applicable) (see Table 1 for abbreviations)
- **dominant_species:** Latin binomials for the dominant species at each site, as reported by site personnel
- **landcover_igbp:** numeric code corresponding to the land cover classification scheme of the International Geosphere-Biosphere Programme, as derived from MODIS remote sensing^39,40^ (http://glcf.umd.edu/data/lc/), where: 0=Water; 1=Evergreen Needleleaf forest; 2=Evergreen Broadleaf forest; 3=Deciduous Needleleaf forest; 4=Deciduous Broadleaf forest; 5=Mixed forest; 6=Closed shrublands; 7=Open shrublands; 8=Woody savannas; 9=Savannas; 10=Grasslands; 11=Permanent wetlands; 12=Croplands; 13=Urban and built-up; 14=Cropland/Natural vegetation mosaic; 15=Snow and ice; 16=Barren or sparsely vegetated; 254=Unclassified; 255=Fill value.
- **wwf_biome:** numeric code corresponding to the biome classification scheme^41^ of the World Wildlife Fund (https://www.sciencebase.gov/catalog/item/508fece8e4b0a1b43c29ca22), where: 1=Tropical & Subtropical Moist Broadleaf Forests; 2=Tropical & Subtropical Dry Broadleaf Forests; 3=Tropical & Subtropical Coniferous Forests; 4=Temperate Broadleaf & Mixed Forests; 5=Temperate Conifer Forests; 6=Boreal Forests/Taiga; 7=Tropical & Subtropical Grasslands, Savannas & Shrublands; 8=Temperate Grasslands, Savannas & Shrublands; 9=Flooded Grasslands & Savannas; 10=Montane Grasslands & Shrublands; 11=Tundra; 12=Mediterranean Forests, Woodlands & Scrub; 13=Deserts & Xeric Shrublands; 14=Mangroves.
- **koeppen_geiger**: climate classification according to the Köppen-Geiger system^42^ (http://koeppen-geiger.vu-wien.ac.at/present.htm), where codes denote a Main climate (A=equatorial, B=arid; C=warm temperate; D=snow; E=polar); a precipitation class (W=desert, S=Steppe; f=fully humid; s=summer dry; w=winter dry; m=monsoonal); and a temperature class (h=hot arid; k=cold arid; a=hot summer; b=warm summer; c=cool summer; d=extremely continental; F=polar frost; T=polar tundra).
- **site_acknowledgments**: data end users are asked to include this text, which has been provided by site collaborators, in publications and presentations that make use of data for this site.

### Data record 2

Data Record 2 contains (1) the “ROI list files”, which detail the date and time range over which each binary image mask was applied in processing the image data for a site; (2) the binary “image mask files”, which delineate the ROI over which the image analysis was conducted; and (3) sample images for each image mask file. With (1) and (2), which we consider as essential metadata, the data sets presented in Data Record 3 can be reproduced from the original image files.

The naming convention for the ROI list files in Data Record 2 is as follows:

- <**sitename**>_<**veg_type**>_<**ROI_ID_number**>_roi.csv

Where **sitename** is the name of the camera site, as listed in the metadata contained in Data Record 1 (e.g., “coweeta”), **veg_type** is a two-letter abbreviation identifying the dominant vegetation within the ROI, e.g. DB for deciduous broadleaf trees (see Table 1), and **ROI_ID_number** is a numeric code that serves as a unique identifier to distinguish between multiple ROIs of the same vegetation type at a given site (0001 for the first ROI list, 0002 for the second, etc.).

A sample ROI list file (coweeta_DB_0001_roi.csv) is as follows:[Boxed-text bx1]

The first 13 lines (beginning with #), document the provenance of the ROI list, and contain a brief description of the vegetation that is delineated by the associated image masks.

Line 14 lists the column headers for the mask entry rows. The mask entries begin on line 15. For this site there was one minor change in the field of view, so there are two ROI mask entries. Any additional field of view changes would result in additional rows (mask entries) being appended to the file. Note that as described in Methods, if the field of view shift is too large or if there are other exogenous events that necessitate distinguishing between the resulting data sets, a new ROI list (e.g., coweeta_DB_0002_roi.csv) would be created for the site.

For each mask entry, the data fields are:

- **start_date** (format: YYYY-MM-DD, where MM=01-12 and DD=01-31)
- **start_time** (format: hh:mm:ss, where hh=00-23, mm=00-59, ss=00-59)
- **end_date** (format: same as for start_date)
- **end_time** (format: same as for start_time)
- **mask_file**: the filename for the 8-bit TIFF mask file with black for the ROI and white for the region to exclude from calculations
- **sample_image**: the filename for a sample image in the date range

Note that only images within the date and time ranges (from **start_date** and **start_time** to **end_date** and **end_time**) listed are included in the processed data set generated from this list. For **end_date**, the date code 9999-12-31 is used to keep the processing open-ended.

The naming convention for the image maskfiles is:

- <**sitename**>_<**veg_type**>_<**ROI_ID_number**>_<**mask_index**>.tif

Here, the **mask_index** matches the entry number in the list (01 for the first entry, 02 for the second entry, etc.). The image mask files are stored in the TIFF image format (.tif) because of the flexibility that this offers, and because of compatibility with the python PIL library.

Sample images for each mask file have the same naming convention but terminate in a .jpg extension:

- <**sitename**>_<**veg_type**>_<**ROI_ID_number**>_<**mask_index**>.jpg

### Data Record 3

Data Record 3 contains time series of ROI colour statistics extracted from the entire image archive (hence “all-image” time series) for each site, for a given ROI, using the ROI list files and image mask files contained in Data Record 2. The naming convention for the text files in Data Record 3 is as follows, with **sitename**, **veg_type**, and **ROI_ID_number** the same as for Data Record 2:

- <**sitename**>_<**veg_type**>_<**ROI_ID_number**>_roistats.csv

These time series have not been filtered, and each data row in the file corresponds to an individual image in the archive. An example file is as follows; for display purposes the lines have been broken with a “\” character:[Boxed-text bx2]

The first 16 lines (beginning with #) contain basic metadata. Line 4 contains the **sitename**, identical to that in the filename, while lines 5 (**veg_type**) and 6 (**ROI_ID_number**) and identify the ROI list. Site location (latitude and longitude in decimal degrees, and elevation in m above sea level; lines 7-9) and UTC offset (line 10) have been extracted from the site metadata contained in Data Record 1. Line 11 indicates whether images have been re-resized to common dimensions (to match the size of the mask file) prior to analysis (for some Type III cameras, the image size varies over time, even as the field of view of the camera is constant; resizing on the fly allows for a consistent region of interest to be analysed without requiring new mask images to be generated). Lines 12-15 document the provenance of the datafile.

Line 17 lists the column headers for the data rows. The data rows begin on line 19, and for each data row (corresponding to an individual image in the archive) the data fields are:

- **date**: local date (format: YYYY-MM-DD)
- **local_std_time**: local standard time (format: hh:mm:ss)
- **doy**: day of year
- **filename**: image filename (format: <sitename>_<YYYY_MM_DD>_<hhmmss>.jpg)
- **solar_elev**: solar elevation angle, in degrees
- **exposure**: image exposure (StarDot cameras only; NA denotes missing values)
- **mask_index**: mask number in the image mask sequence
- **gcc**: mean green chromatic coordinate (*G*_cc_) over the ROI
- **rcc**: mean red chromatic coordinate (*R*_cc_) over the ROI
- **r_mean**: mean red channel DN over the ROI
- **r_std**: standard deviation (across pixels) of red channel DN over the ROI
- **r_5_qtl, r_10_qtl, r_25_qtl, r_50_qtl, r_75_qtl, r_90_qtl, r_95_qtl**: the 5, 10, ..., 90, 95^th^percentile values (across pixels) of the red channel DN over the ROI
- **g_mean, g_std, g_5_qtl, g_10_qtl, g_25_qtl, g_50_qtl, g_75_qtl, g_90_qtl, g_95_qtl**: same as above for green channel
- **b_mean, b_std, b_5_qtl, b_10_qtl, b_25_qtl, b_50_qtl, b_75_qtl, b_90_qtl, b_95_qtl**: same as above for blue channel
- **r_g_cor**: correlation coefficient (across pixels) between red channel DN and green channel DN, over the ROI
- **g_b_cor**: correlation coefficient between green channel DN and blue channel DN, over the ROI
- **b_r_cor**: correlation coefficient between blue channel DN and red channel DN, over the ROI

### Data Record 4

Data Record 4 contains the 1-day and 3-day summary product files derived from the higher-frequency all-image time series that are contained in Data Record 3. The naming convention for the summary product files is as follows, with **sitename**, **veg_type**, and **ROI_ID_number** the same as for Data Record 2:

- <**sitename**>_<**veg_type**>_<**ROI_ID_number**>_1day.csv
- <**sitename**>_<**veg_type**>_<**ROI_ID_number**>_3day.csv

An example 1-day summary product file is as follows; for display purposes, the lines have been broken with a “\” character. The format of the 1- and 3-day summary files is identical and, as noted in the Methods, the only difference between the two file types is the 1- vs. 3-day aggregation period.[Boxed-text bx3]

The first 24 lines (beginning with #) contain basic metadata. Lines 4 through 10 are identical to those in the all-image time series file from which the summary product files are derived. Line 11 is not used in the datasets reported here, but our current processing workflow allows for the specification of an image count threshold for processing to occur (i.e., if for a given period of aggregation, there are insufficient images available, then only results for the midday image, if applicable, would be reported). Line 12 gives the number of days that have been aggregated in producing the file, which is 1 day for Data Record 4 and 3 days for Data Record 5. Line 13 reports the solar elevation filter that was used in processing (10° in the current dataset). Lines 14 and 15 are not used here, but our current processing workflow allows for the specification of time-of-day window (i.e., images outside of the window would be excluded from the processing). Lines 16 and 17 report the values that were used for the “too dark” and “too bright” quality control filters, which are by default set to DN 100 and 665, respectively. Lines 18-23 document the provenance of the datafile.

Line 25 lists the column headers for the data rows. The data rows begin on line 24, and for each data row the data fields are:

- **date**: local date at the middle of the aggregation period (1-day or 3-day) (format: YYYY-MM-DD)
- **year**: calendar year of the above date (YYYY).
- **doy**: day of year for the above date. The date/doy values chosen are for fixed days-of-year (i.e., for the 3-day summary files these will always be doy=2, 5, 8, etc.). Note that for the 3-day summary files, data for the final aggregation period (doy=365) is calculated using only two days of data in non-leap years.
- **image_count**: the number of images passing the selection criteria, as described in Methods
- **midday_filename**: the filename of the image which is closest to 12:00 noon (the “midday image”), local standard time, on the middle day of the aggregation period
- **midday_r**: mean red channel DN over the ROI, for the midday image
- **midday_g**: mean green channel DN over the ROI, for the midday image
- **midday_b**: mean blue channel DN over the ROI, for the midday image
- **midday_gcc**: the mean *G*_CC_ over the ROI, for the midday image
- **midday_rcc**: the mean *R*_CC_ over the ROI, for the midday image
- **r_mean**: the mean value (for all images passing the selection criteria) of the mean (by image) red channel DN over the ROI
- **r_std**: the standard deviation (for all images passing the selection criteria) of the mean (by image) red channel DN over the ROI
- **g_mean, g_std, b_mean, b_std**: as above for **r_mean** and **r_std**, but for the green channel and the blue channel
- **gcc_mean**: the mean value (for all images passing the selection criteria) of the mean (by image) *G*_cc_ over the ROI
- **gcc_std**: the standard deviation (for all images passing the selection criteria) of the mean (by image) *G*_cc_ over the ROI
- **gcc_50**, **gcc_75**, **gcc_90**: the 50^th^, 75^th^ and 90^th^ percentile value(for all images passing the selection criteria) of the mean (by image) *G*_CC_over the ROI
- **rcc_mean**, **rcc_std**, **rcc_50**, **rcc_75**, **rcc_90**: as above for *G*_CC_, but for *R*_CC_
- **max_solar_elev**: the maximum solar elevation angle for all images passing the selection criteria
- **snowflag**: a citizen-science based evaluation of the presence of snow in the midday image, as described in Methods. The **snowflag** is coded as follows: 1=bad or obscured image; 2=no snow in image; 3=snow on ground (used for non-tree sites); 4=snow on ground only (used for treed sites); 5=snow on trees (and ground; used for treed sites). If the midday image was not evaluated, a value of NA is assigned.
- **outlierflag_gcc_mean, outlierflag_gcc_50, outlierflag_gcc_75, outlierflag_gcc_90**: the **outlierflag**, which is determined separately for the gcc_mean, gcc_50, gcc_75, and gcc_90 time series, can either take on a value of 0 (indicating good data), or 1 (indicating an outlier)
- **smooth_gcc_mean, smooth_gcc_50, smooth_gcc_75, smooth_gcc_90**: the smoothed and/or interpolated value of G_cc_ from the final iteration (i.e. with outliers removed) of the spline fitting process
- **smooth_rcc_mean, smooth_rcc_50, smooth_rcc_75, smooth_rcc_90**: as above for *G*_CC_, but for *R*_CC_
- **smooth_ci_gcc_mean, smooth_ci_gcc_50, smooth_ci_gcc_75, smooth_ci_gcc_90**: the (one-sided) width of the 95% confidence interval around the smoothed *G*_CC_ values
- **smooth_ci_rcc_mean, smooth_ci_rcc_50, smooth_ci_rcc_75, smooth_ci_rcc_90**: as above for *G*_CC_, but for *R*_CC_
- **int_flag**: to assist with identification of long gaps in the data record, the interpolation flag is set to 1 during a gap of 14 days or more.

Dates for which there are no images (or none passing the selection criteria) have empty fields, although smoothed values of *G*_CC_ and *R*_CC_ are reported, along with their uncertainties. When a particular value is missing or cannot be calculated it is given a “no data” value of NA.

### Data Record 5

Data Record 5 contains the transition date estimates for the start of each “greenness rising” stage and end of each “greenness falling” stage, derived separately from the 1-day and 3-day summary files contained in Data Record 4.

The naming convention for the transition date estimate files in Data Record 5 is as follows, with **sitename**, **veg_type**, and **ROI_ID_number** the same as for Data Record 2:

- <**sitename**>_<**veg_type**>_<**ROI_ID_number**>_1day_transition_dates.csv
- <**sitename**>_<**veg_type**>_<**ROI_ID_number**>_3day_transition_dates.csv

[Boxed-text bx4]

The first 16 lines (beginning with #) contain basic metadata. Lines 4 through 6 are identical to those in the summary product file from which the transition date file is derived. Line 7 gives the number of days that have been aggregated in producing the file, which is either 1 or 3 days. Lines 8 and 9 define the first and last years for which the transition dates are calculated. Lines 10 and 11 document the provenance of the datafile. Lines 12-15 report goodness-of-fit statistics (in terms of RMSE, the root mean squared error) for the spline curves from which the transition dates are extracted.

Line 17 lists the column headers for the data rows.The data rows begin on line 18, and for each data row, corresponding to a single “greenness rising” or “greenness falling” stage, the data fields are:

- **sitename:** the name of the camera site
- **veg_type**: a two-letter abbreviation identifying the dominant vegetation within the ROI (see Table 1)
- **roi_id**: a numeric code (**ROI_ID_number)** to distinguish between multiple ROIs of the same vegetation type at a given site
- **direction**: indicates whether the reported transition dates correspond to a “greenness rising” or “greenness falling” stage. Note that there may be more than one rising/falling cycle per calendar year, and a single rising or falling stage may cut across years.
- **gcc_value:** indicates whether the transition dates are calculated from gcc_mean, gcc_50, gcc_75 or gcc_90 time series (a typical file will include dates calculated for each of these)
- **transition_10, transition_25, transition_50**: the extracted transition dates (format YYYY-MM-DD) for each “greenness rising” or “greenness falling” stage, corresponding to 10%, 25% and 50% of the *G*_CC_ amplitude of that stage.
- **transition_10_lower_ci, transition_25_lower_ci, transition_50_lower_ci, transition_10_upper_ci, transition_25_upper_ci, transition_50_upper_ci**: dates (format YYYY-MM-DD) corresponding to the lower and upper, respectively, 95% confidence intervals on the extracted transition dates (10%, 25%, and 50% of the *G*_CC_ amplitude)
- **threshold_10, threshold_25, threshold_50**: the threshold values of *G*_CC_ used to identify transition dates
- **min_gcc,max_gcc:** the baseline (dormant-season minimum) and peak (active-season maximum) *G*_CC_ values, calculated from the fitted spline, as used to derive the *G*_CC_ amplitude. These values can be used to normalize the *G*_CC_ time series to a 0-1 scale, if desired.

## Technical Validation

Here we discuss analyses to (1) assess the impact of camera choice on the seasonal patterns of canopy colour derived from digital camera imagery, and the transition dates extracted from those colour indices; (2) characterize the spectral sensitivity and sensor response of the standard camera used at the majority of PhenoCam sites, and evaluate the long term stability of the sensor; (3) quantify the consistency of the image colour balance in response to changing weather conditions and illumination geometry by analysis of a reference panel; (4) demonstrate the importance of accounting for field of view shifts via adjusted ROI masks; and (5) evaluate the technical quality of the data presented here by comparison against other types of ground measurements and remotely sensed observations.

Together, the data sets and validation studies we describe demonstrate that: (1) conventional, visible-wavelength digital cameras have the necessary capabilities—colour channel sensitivity to appropriate wavelengths, stable colour balance, insensitivity to exposure differences, stability over multiple years, and high signal-to-noise ratio—to reliably detect seasonal changes in the structure and colour dynamics of vegetation; (2) these capabilities are highly robust to differences in the specifics of camera design and in-camera image processing; and (3) camera phenology data are consistent with other phenology data, including ground observations, near-surface radiometry, and satellite remote sensing. These analyses thus provide strong evidence in support of the overall quality and utility of data derived from PhenoCam imagery.

### Camera selection

A wide variety of different models of cameras are currently being used for phenological monitoring, both within the PhenoCam network and by other researchers^14,15,43,44^. Differences in the physical attributes of a camera’s design (perhaps most importantly, the size, quality, and dynamic range of the imaging sensor) and within-camera image processing (all of the steps that occur between photons landing on the imaging sensor and the production of a digital image) mean that any two cameras may produce different-looking images even when observing exactly the same scene. However, one previous study^15^ compared the trajectory of autumn senescence in an oak-dominated temperate deciduous forest, as characterized by eleven different digital cameras, all installed next to each other and aimed at the same region of the canopy. This analysis showed that although the images from different cameras looked different, particularly with regard to colour balance and contrast, the transition dates derived from each camera’s image time series were largely consistent with each other (with the exception of imagery from an inexpensive webcam designed for indoor use), and for the most part were constrained to within a day or two of the mean.

These findings are further substantiated by a long-term experiment we have been conducting in a maple-beech-birch temperate deciduous forest. At that site, two different cameras (Type II site “bartlett”: model 211, Axis Communications, Lund, Sweden^17^; Type I site “bartlettir”: model NetCam SC 1.3 MP IR, StarDot Technologies, Buena Park, CA, USA^15^) have been mounted adjacent to each other since the start of the 2008 growing season. The Axis camera has a smaller sensor and a narrower field of view than the StarDot camera; for this analysis, we have defined ROIs to be as similar as possible between the two cameras.

**Figure 3:**
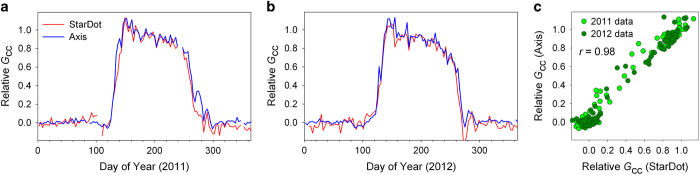
Comparison of seasonal patterns of canopy greenness, expressed as relative *G*_cc_ (see text), derived from imagery from two different camera brands/models (StarDotNetCam SC IR and Axis 211). The cameras were installed overlooking a temperate deciduous forest canopy (sites “bartlett” and “bartlettir”: Bartlett Experimental Forest, New Hampshire). (**a**) Data for 2011; (**b**) Data for 2012; (**c**) bivariate scatter plot showing linear relationship, negligible seasonal hysteresis, and consistent patterns between years.

For these two cameras, time series from two sample years, 2011 and 2012, are shown in Fig. 3 (here, to accommodate absolute differences in *G*_cc_ between the two cameras, *G*_cc_ has been linearly re-scaled each year to “Relative *G*_cc_”, where mean *G*_CC_ over the dormant season—after day 290 and before day 110—is set to 0, and mean peak summer *G*_CC_—after day 150 and before day 180—is set to 1). The coherence between the two time series—particularly with regard to the timing of spring green-up, the pronounced late-spring “green spike”^19^, the gradual summer green-down, and the more rapid autumn green-down—is visually apparent (Fig. 3a,b). The linear correlation (Fig. 3c) between the two time series is very strong (*r*=0.98).

Over the seven years (2008-2014) of this experiment, transition dates derived from the Axis camera imagery are in excellent agreement with those derived from the StarDot camera imagery. For example, using dates of 50% amplitude during the “rising greenness” and “falling greenness” stages, in spring the dates from the two cameras agree to with 0.4±1.4 d (mean±1 standard deviation, *N*=7 years; pairwise correlation of dates, *r*=0.97) while in autumn the dates from the two cameras agree to within 1.3±2.4 d. A low pairwise correlation of dates in autumn (*r*=0.28) can be attributed to the much lower interannual variability in autumn transition dates (1 SD=1-2 d) derived from *G*_CC_, compared to spring transition dates (1 SD=5-7 d). However, the timing of peak autumn colours (determined by analysis of *R*_cc_^15,18,20^), is more variable from year-to-year, and between the two cameras is in agreement to within 1.0±0.7 d (pairwise correlation of dates, *r*=0.96). Thus, we argue that both the seasonal patterns of canopy colour extracted from the camera imagery, and the transition dates derived from those colour indices, are highly robust to differences in the specifics of camera design and in-camera image processing.

### Spectral sensitivity and sensor response

All of the cameras used here produce three-colour images by means of a colour filter array, called a Bayer filter mosaic, which consists of a specific arrangement of red, green and blue filters on the surface of the imaging sensor. By recording pictures of materials with different spectral signatures (400-1,000 nm) under standardized illumination conditions in the lab, one previous study^45^ characterized the spectral sensitivity of the standard StarDot NetCam SC camera, used at the majority of PhenoCam sites, and all Type I sites. This analysis showed that the blue channel DN was best correlated (*r*=0.92) with object reflectance from 430–515 nm, while green channel DN was best correlated (*r*=0.94) with object reflectance from 510–570 nm and red channel DN was best correlated (*r*=0.96) with object reflectance from 575–710 nm. Thus, each of the three colour channels is sensitive to the expected spectral range (i.e., the green channel is sensitive to green and yellow wavelengths), and none of the three colour channels is particularly sensitive to wavelengths outside the expected spectral range (i.e., the green channel has little sensitivity to blue or red wavelengths). This means that there is minimal waveband overlap among the red, green and blue colour channels. These results are consistent with a recent study in which the spectral response of the imaging sensor in the StarDot NetCam SC was characterized using a monochromator^43^.

We used a previously developed approach^46^—commonly known as Debevec’s method, after its developer—to characterize the response function of the imaging process for 10 StarDot cameras, all of the same standard model (NetCam SC). This analysis included older cameras (purchased in 2008) that were previously deployed in the field for 5 years or more, newer cameras that were previously deployed for only a couple of years (purchased in 2011), and brand new cameras that had never left the laboratory (purchased in 2015). Our objective was to determine how similar the sensor response function was across cameras, and whether older cameras showed signs of sensor degradation that might adversely influence the data presented here.

Debevec’s method uses repeated images of the same scene (here a colour target), each taken under constant illumination with a different integration time, to recover the response function. The method assumes reciprocity, i.e. the total amount of light energy recorded by the sensor is determined by the product of the sensor integration time and the light intensity, which implies that the same image can be obtained with a longer integration or greater light intensity^46^.

To ensure that our process was repeatable, we built a rigid mount with two 50 cm adjustable swing arms attached to a back plate. The camera and a standard 6.2 mm lens (which was reserved for sensor characterization) was mounted on one arm, and a 50 W halogen diffuse light source (ProLamp, Analytical Spectral Devices Inc., Boulder CO) on the other arm. The arms were set at approximately +5° and —5° from the back plate’s perpendicular axis, and a 24 patch colour target (ColorChecker Classic, X-Rite Inc., Grand Rapids MI) was affixed to the back plate, centred within the camera’s field of view. The whole apparatus was placed inside a cardboard box to shield it from stray light. We then recorded 9 sequential images, with integration times increasing from 1/9600 s to 1/160 s. The centre of each colour patch was selected manually by visual inspection of the fourth image (integration time=1/960 s), and a 51×51 pixel neighbourhood centred on each centre pixel was used to calculate the mean colour for each patch on each image. Calculating a mean colour for the centre of each patch minimized the impact of any JPEG compression artefacts. The colour data were then processed in MATLAB to produce curves of camera DN (*y* axis) versus the natural logarithm of exposure (the product of integration time and irradiance) (*x* axis), according to the code provided with Debevec’s publication.

The analysis indicated that the different NetCam sensors had similar response functions, regardless of sensor age and previous deployment conditions. We found that for each colour channel, the recovered sensor response was similar across all 10 NetCam SC cameras that we characterized. In addition, we could distinguish these responses from the response obtained when camera settings were changed to non-standard values, or with a different camera model. For all three channels, we found a smooth and monotonically increasing relationship between exposure and digital number, with relatively little scatter in the range of DN 35 to DN 220. Minor outliers were found to be associated with colour patches that approached saturation in one or more of the colour channels of the image with the longest integration time.

**Figure 4:**
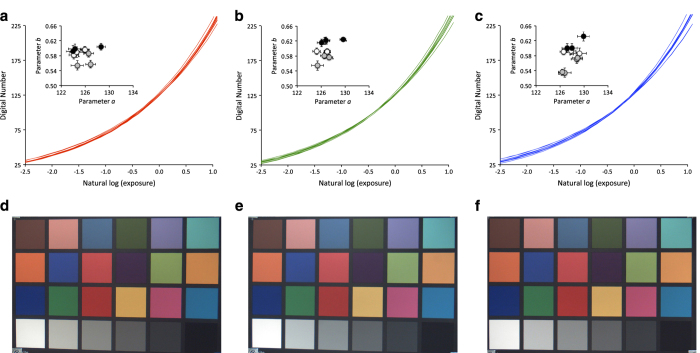
Evaluation of differences in imaging sensor response characteristics across cameras of varying ages. The sensor response is characterized in terms of the digital number (y axis) produced for a given exposure (x axis, natural logarithm). (**a**-**c**) sensor response functions (plotted as *y*=*a* * exp (*b* * x), and response function parameters (inset) for 9 StarDot cameras of varying ages (whitecircles: 2008 cameras, grey circles: 2011 cameras, black symbols: 2015 cameras). Each color channel is plotted separately: (**a**) red channel; (**b**) green channel; (**c**) blue channel. Note that variation among cameras is minimal for DN <150. (**d**–**f**) sample images, at a constant exposure, from cameras of varying ages: (**d**) 2008 camera; (**e**) 2011 camera; (**f**) 2015 camera.

For each of the three colour channels on each camera, we characterized the response function (*x* is the natural log of exposure, *y* is the corresponding DN output) by means of an exponential relationship (Equation 2),

(2)y=a*exp(b*x)+ε

where *a* and *b* are fitted parameters and ε is the regression residual. For each colour channel, the fitted response functions were similar among cameras in terms of overall shape and position (main plot Fig. 4a–c). And, for a given exposure, there was relatively little variation among cameras in the resulting DN output. Indeed, the variation among cameras was minimal below about DN 150 (corresponding approximately to a natural log of exposure of 0.5). Variation among cameras tended to be more pronounced above these exposure / DN output values, corresponding to increasing steepness of the response function. Furthermore, we found that there was no obvious relationship between camera age and either of the fitted parameters *a* or *b* (inset Fig. 4a–c). Consequently, for well-exposed test images, any differences among cameras in the resulting image are barely discernable to the human eye (Fig. 4d–f).

Overall, this exercise (1) supports our method of excluding images that are too bright (mean total DN > 665) or too dark (mean total DN < 100) from further processing (note that within a typical forest canopy ROI, the mean total DN is roughly 150–250, or DN 50–80 per channel), and (2) suggests minimal degradation of the imaging sensor even after many years of field use, and good consistency across different cameras of the same model. These findings enhance our confidence in the phenological patterns observed across sites.

We also evaluated the long-term stability of the imaging sensor by looking for trends (i.e. drift) in the winter baseline *G*_cc_ value for those sites with deciduous broadleaf (DB) ROIs and long-term (5+ year) records. Our assumption is that while the summertime *G*_cc_ might exhibit trends due to changes in the amount of leaf area (potentially related to forest succession), or the colour of individual leaves (potentially related to nutrient availability, e.g. nitrogen deposition, or stress factors, e.g. insects and other pests/pathogens), there is no *a priori* reason to expect the colour of leafless branches to change substantially over time. This approach is conceptually similar to one adopted in a previous study, where sensor degradation over a 6 year period was quantified using shifts in the colour of snow and sky^47^. Across all sites (*N*=41 sites, with a total of more than 250 years of data), the median trend in winter baseline *G*_cc_ (here, defined as mean *G*_CC_ from day 345 to day 60 in the following year) was +0.0005 units y^-1^(25^th^ percentile, -0.0003 units y^-1^; 75^th^ percentile +0.0015 units y^-1^). At 6 sites there was a significant (*P*<0.05) trend towards rising wintertime *G*_CC_, while at 1 site there was a significant trend towards falling wintertime *G*_CC_. The “morganmonroe,” “harvard” and “proctor” sites are typical of those with significant trends: at each of these, there is very gradual but consistent increase in the wintertime *G*_CC_, but even accumulated over a whole decade, the trends are small enough to amount to a baseline shift of only 0.01 *G*_CC_ units (or less) over 10 years. Two exceptions are the “nationalcapital” (trend=+0.0054 units y^-1^, *P*<0.01) and “umichbiological2” (trend=+0.0041 units y^-1^, *P*=0.02) sites, for which visual inspection of these time series suggests that the trends are driven by shifts in camera field of view that, despite our best efforts, are not fully captured by the new ROI masks.

To put the above trends in perspective, the mean day-to-day variability (1 SD) in mid-day wintertime *G*_cc_ at the long-term sites examined is 0.0036±0.0027 units (mean±1 SD, across sites), about a mean winter value of 0.3404±0.0194 units (mean±1 SD, across sites). Thus, we conclude that the observed trends, even when statistically significant, are generally small relative to the intrinsic day-to-day variability in *G*_CC_.

### Reference panel analysis

We used a grey Spectralon (LabSphere, North Sutton, NH) diffuse reflectance standard, positioned within the camera field of view at the “harvardbarn” and “howland1” sites, to evaluate the consistency of the image colour balance in response to changing weather conditions and illumination geometry. We studied variation in reference panel *G*_cc_ across the entire calendar year (all images with solar elevation greater than 5°, but excluding those where the mean [red DN + green DN + blue DN] across the reference panel was < 200 or > 565). This analysis builds on that presented in a previously-published study which used a painted grey panel to evaluate the day-to-day variation in image colour balance: in that analysis^18^, although the overall DN of each colour channel was highly variable from day-to-day, the colour balance of the reference panel, as characterized by chromatic coordinates, was shown to be very stable.

Our new analysis confirms the previous results. For harvardbarn (*N*=13,223 images from calendar year 2014), the overall standard deviation of reference panel *G*_cc_ was 0.0042 *G*_cc_ units—by comparison, the seasonal amplitude of *G*_cc_ for deciduous trees in the camera’s field of view was roughly 25 times larger. Variation in reference panel *G*_CC_ was accounted for partially (25%) by illumination geometry (solar elevation and azimuth), and partially (an additional 25%) by a seasonal cycle (which may in part result from light reflected off the canopy striking the reference panel). The residual not accounted for by these two factors had a standard deviation of 0.0029 *G*_cc_ units.

For howland1 (*N*=2,830 images from calendar year 2014), the overall standard deviation of reference panel *G*_cc_ was 0.0040 *G*_cc_ units, and thus very similar to that for harvardbarn. Variation in howland1 reference panel *G*_cc_ was accounted for partially (25%) by illumination geometry and to a lesser degree (an additional 7%) by a seasonal cycle. The residual not accounted for by these two factors had a standard deviation of 0.0033 *G*_cc_ units.

These results imply that an approximate 99% (2.5σ) confidence interval on individual *G*_cc_ values is, conservatively, about 0.0100 (=2.5 x 0.0040) *G*_cc_ units.

Thus, analysis of reference panel data serves to give high confidence in both (1) the observed seasonal signal, and (2) the minimal impact of day-to-day variability in lighting.

### Accounting for camera field of view shifts

Camera field of view shifts have the potential to significantly impact data quality, if the shift is sufficiently large that the ROI mask ceases to be representative of the target vegetation. Thus, small field of view shifts are generally not problematic for large ROIs and homogeneous vegetation, but large field of view shifts are particularly problematic for small ROIs and heterogeneous vegetation. Large field of view shifts are common for some sites (particularly Type III sites) but rare at other sites. The number of identified field of view shifts for an ROI corresponds to the number of image mask files contained in Data Record 2.

**Figure 5:**
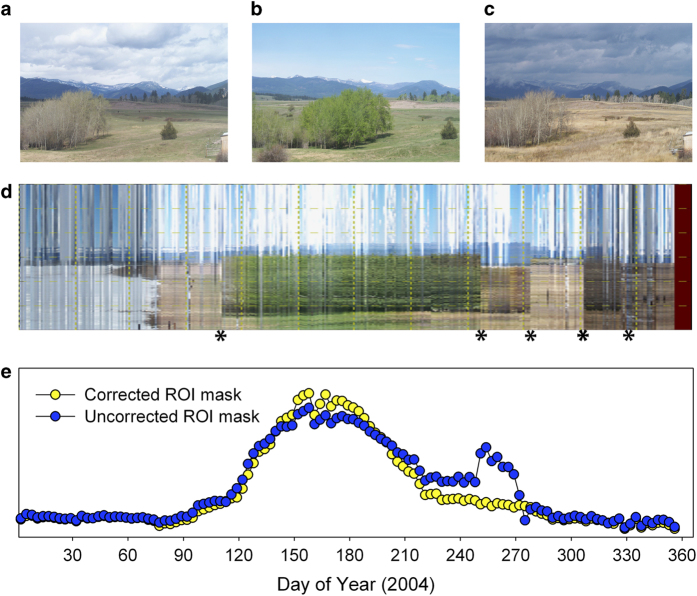
Example of impact of field of view shifts on camera imagery and derived *G*_CC_ time series for a site with mixed vegetation. Field of view shifts in imagery (site: “monture”, Lolo National Forest, Ovando, Montana, year: 2004) were detected on April 20 (day 111), September 8 (day 252), October 5 (day 278), November 3 (day 308), and November 23 (day 327). (**a**-**c**) Sample images are from April 16, May 6, and October 23. Note the changing position of the small, evergreen conifer right of centre. (**d**) Composite image, in which field of view shifts are easily identified. Asterisks along the bottom of the image denote the dates of the 5 shifts that were detected. (**e**) Time series of *G*_CC_, the green chromatic coordinate, showing the difference in the inferred grassland seasonality when the region of interest (ROI) mask is corrected for shifts in the field of view. The corrected mask includes only grassland vegetation, while the uncorrected mask includes a mix of grassland as well as deciduous or evergreen tree foliage. Artifacts are visually apparent in the data derived from the uncorrected ROI mask. The y-axis ticks and labels have been omitted from (**e**) to facilitate alignment with (**d**). The y-axis range is approximately 0.32 to 0.44 *G*_CC_ units.

The impact of field of view shifts is documented in Fig. 5, in which we compare the *G*_CC_ time series that are produced when the ROI masks are not corrected for field of view shifts, and when they are corrected for field of view shifts. This example is for the site “monture”, a Type III site. Although automated methods to detect these shifts are needed in order to more efficiently process camera imagery, this example demonstrates that data quality is greatly improved when such shifts can be identified and accounted for in processing.

### Comparison with other types of data

A number of recent studies have compared canopy greenness measures derived from digital camera imagery (and associated seasonal transition dates) with a variety of other measurements and observational data. We review these here, and also present some new analyses. We begin by presenting analyses that relate to the biological interpretation of seasonal changes in *G*_cc_, and then assess the degree to which PhenoCam data are consistent with seasonal patterns inferred from near-surface radiometric measurements, and from satellite remote sensing.

#### Biological interpretation

The biological relevance of seasonal changes in canopy *G*_cc_ and the green-up and green-down transition dates derived from *G*_cc_ has been previously assessed in a number of ways. For example, two studies have exhaustively investigated the factors underlying the seasonal trajectory of forest canopy greenness, specifically with regard to changes in leaf-level traits, changes in canopy structure, and changes in camera-derived canopy greenness^19,48^. A nonlinear mixing model has been used to show how canopy-level *G*_cc_ is directly related to the amount of leaf area present (i.e., LAI, leaf area index), the colour of individual leaves (which was measured on a flatbed scanner), and the colour of the (leaf-free) background (i.e., branches and forest floor)^19^. The resulting model was able to successfully simulate the seasonal trajectory of canopy-level *G*_cc_, including the pronounced *G*_cc_ “spike” in late spring. The model also showed that canopy-level *G*_cc_ is relatively insensitive to changes in LAI above intermediate index values (≈2.5 or higher).

**Figure 6:**
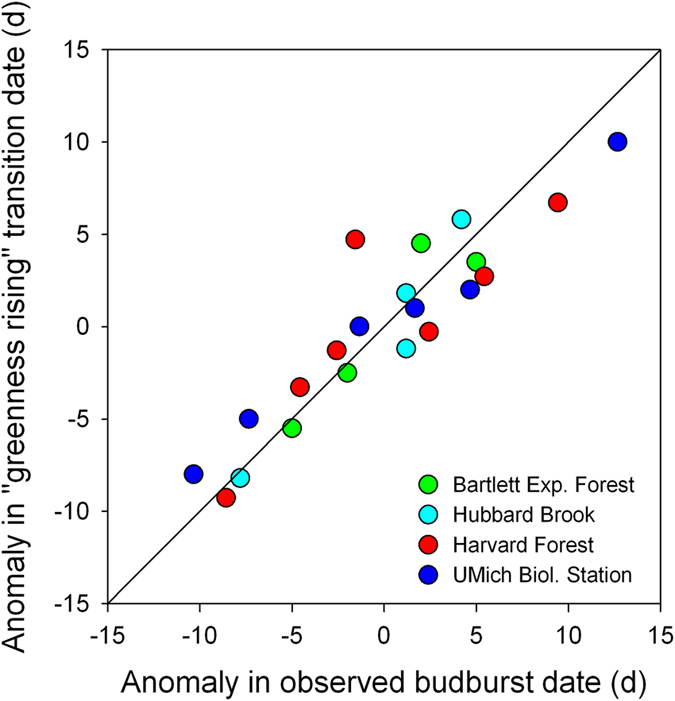
Comparison of direct observation of budburst date by a human observer with “greenness rising” spring transition date (transition_25) derived from camera *G*_CC_. All data have been converted to anomalies from the site mean to account for differences in ground observation protocol across sites. The solid line is the 1:1 line.

Transition dates derived from *G*_cc_ have also been compared with ground observations of deciduous forest phenology (leaf development, autumn coloration, and leaf drop) made by a human observer^19^. Here, we repeat the analysis of spring budburst and leaf development using more years of data, and data from three additional research sites. At both the Harvard Forest LTER (phenocam site: “harvard”, *N*=7 y of ground data) and the Hubbard Brook LTER (phenocam site: “hubbardbrook”, *N*=5 y of ground data) sites, observations of phenology have been made on a set of marked trees by the same two observers for more than 20 y^49^. At the University of Michigan Biological Station (site: “umichbiological”, *N*=6 y of ground data), ground observations of phenology on a set of marked trees began in 1999. At Bartlett Experimental Forest (site: “bartlettir”, *N*=4 y of ground data) continuous, upward-looking hemispherical photos have been recorded since 2013 and permit assessment of phenology independent of the PhenoCam imagery. Because protocols for defining spring onset are site-specific, and because of potential mismatches between the species mixture in the camera field of view and the individuals that were observed, offsets from the 1:1 line also tended to vary among sites. At Bartlett (mean offset±1 SD, 1.5±1.7 d), Harvard (0.7±3.3 d), and Hubbard Brook (-0.6±1.5 d), the camera-derived spring transition date (threshold_25%) occurred at approximately the same time as the observer-identified date. By comparison, at the University of Michigan Biological Station the camera-derived spring transition date generally occurred *after* the observer-identified date (mean offset, 6.7±2.3 d). However, when dates were converted to anomalies from the site mean, the correlation between camera-derived and human-observed spring dates was extremely strong (*r*=0.92, *N*= 22; Fig. 6) and the Type II regression slope was not significantly different from 1.0. (*P* > 0.10).

Using a slightly different approach, one recent study compared seasonal transition dates from *G*_cc_ time series against visually assessed dates, as determined by a human observer looking at the same imagery^20^. This analysis used more than 70 site-years of imagery and data from PhenoCam. In spring, the start of green-up (which varied by more than 7 weeks across sites) was linearly correlated with the visually assessed date when “the majority of trees started leafing out” (*r*=0.89, RMSD=7 d, bias=0 d). In autumn, the middle of green-down (which varied by more than 8 weeks across sites) was linearly correlated with the visually assessed date when “the canopy exhibited the brightest fall colours” (*r*=0.89, RMSD=9 d, bias=–6 d). Thus, the transition dates presented here show a high degree of concordance to visually detectable changes in vegetation state. These results are consistent with those of other recent studies^29,43^.

At the ecosystem scale, and across a wide range of ecosystem types, measures of canopy greenness derived from digital imagery have also been shown to correlate well with the seasonal dynamics of gross photosynthesis, as derived from eddy covariance measurements of surface-atmosphere CO_2_ flux^18,26,50–53^. Thus, information about the state of the canopy, and its level of physiological activity, can be inferred from the PhenoCam data presented here.

#### Near-surface radiometric measurements

A previous study^45^ compared *G*_cc_ derived from camera images recorded in the field with that calculated from calibrated, narrow-band (blue: 470±20 nm, green: 557±25 nm, red: 605±35 nm) radiometric sensors (Model 1850, Skye Instruments, Llandrindod Wells, UK) overlooking the same forest canopy (temperate deciduous forest; site: “harvardbarn”). This analysis showed that the seasonal patterns were nearly identical between camera *G*_cc_ and radiometer *G*_cc_, in particular the steep rise in *G*_cc_ from day 114 to day 143, the gradual decline in *G*_cc_ from day 143 to day 250, and the more rapid decline beginning on day 250 and continuing through day 300. On days where there were data from both instruments, there was a strong linear relationship between camera *G*_cc_ and radiometer *G*_cc_ (*r*=0.95, *N*=200). That the agreement between camera *G*_cc_ and radiometer *G*_cc_ is so strong gives us high confidence in our ability to characterize vegetation colour from camera imagery.

**Figure 7:**
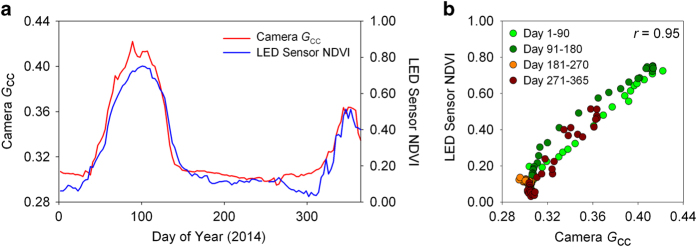
Comparison of camera *G*_cc_ and LED sensor NDVI recorded at a Mediterranean grassland (site “vaira”: Vaira Ranch, California). (**a**) Time series plots showing the parallel evolution of camera *G*_CC_ and LED sensor NDVI over the course of 2014. (**b**) bivariate scatter plot showing moderate seasonal hysteresis (compare day 1-90 vs. day 91-180) in the relationship between camera *G*_cc_ and LED sensor NDVI.

We have conducted similar analyses at other sites. For example, at a Mediterranean grassland (site: “vaira”), there is overlap between the field of view of our camera and the footprint of a spectral reflectance sensor built with light emitting diodes (LEDs)^54^. We compared camera *G*_cc_ with LED sensor measurements of the normalized difference vegetation index (NDVI). NDVI is commonly used in satellite remote sensing studies of vegetation phenology as a measure of green vegetation cover^13^. NDVI differs from *G*_cc_ in that NDVI is calculated from reflectances of both visible (specifically, red) and near infrared wavelengths, whereas *G*_cc_ is based entirely on visible wavebands. Nevertheless, our results indicate an excellent coherence between LED sensor NDVI and camera *G*_cc_ for the year 2014 (Fig. 7a), specifically with regard to the timing and relative magnitude of the green peaks around day 100 and day 350. Overall, there is a strong linear correlation (*r*=0.98) between the two time series (Fig. 7b). The small amount of seasonal hysteresis that is apparent (Fig. 7b) is likely related to inherent differences between the seasonal trajectories of NDVI and *G*_cc_. It could also be the result of slight mismatches between the image ROI that was analysed and the footprint of the LED sensor.

#### Satellite remote sensing

A growing number of studies have compared transition dates derived from PhenoCam time series of *G*_cc_ with transition dates derived from satellite remote sensing^20,21,55,56^. As an example, in one recent paper^20^, the comparison against transition dates derived from satellite remote sensing was similarly strong in both spring (start of green-up: *r*=0.82, RMSD=9 d, bias=1 d; middle of green-up: *r*=0.94, RMSD=6 d, bias=–3 d) and autumn (middle of green-down: *r*=0.85, RMSD=10 d, bias=–7 d; end of green-down: *r*=0.88, RMSD=11 d, bias=–8 d). In light of the substantial statistical uncertainty (at 95% confidence, ≈6–8 d in spring, ≈8–12 d in autumn) associated with deriving phenological transition dates from 8-day satellite data products, and the potential for the vegetation in the camera field of view to be non-representative of the broader landscape, these results indicate remarkably good agreement.

## Usage Notes

We expect that the data presented here will be of use to researchers in a variety of fields, from plant biology and ecosystem ecology to geography and earth system science^8,14^. Among other applications, PhenoCam data can be used (1) as ground observations for evaluation of satellite remote sensing products^20,21,27^; (2) for phenological model development and testing^23,24,52,57^; or (3) to investigate relationships between land cover dynamics and ecosystem function^19,26^. To assist with data exploration and visualization, we have developed an interactive web page (http://explore.phenocam.us/), which enables the user to browse the dataset described here, plot time series and extracted transition dates, and download bundled data sets for individual sites.

We suspect that PhenoCam data will be of greatest scientific value when paired with other types of data. Here we describe some of the other data sets, all publicly available, which we believe may be of use to end-users of PhenoCam data, and the tools (generally written for the R software environment, as bash scripts, or as stand-alone applications) that we have developed to facilitate integration with the PhenoCam database.

For many applications, researchers will need meteorological data to provide context for the seasonal patterns indicated by the PhenoCam time series. Daymet (http://daymet.ornl.gov/), archived and distributed through the ORNL DAAC (Oak Ridge National Laboratory, Distributed Active Archive Center for Biogeochemical Dynamics), is a gridded (1 km x 1 km spatial resolution) weather product that includes, at a daily time step, minimum and maximum temperature, precipitation, humidity, shortwave radiation, snow water equivalent, and day length^36^. Data are available back to 1980 for continental North America south of 52°N latitude. An R script which will take the 1-day summary product data file (i.e., as contained in Data Record 4) for a given site, download the appropriate Daymet data for that site location, and merge the two datasets, is available here: https://github.com/khufkens/daymetr. Historical weather data are also available, for individual sites in NOAA’s (National Oceanic and Atmospheric Administration) surface observation network, through the National Centers for Environmental Information (https://www.ncei.noaa.gov/).

High frequency measurements of the surface-atmosphere exchanges of CO_2_, H_2_O and energy are made, using the eddy covariance technique, at several hundred sites within the AmeriFlux network^58^. Many of these sites are also members of the PhenoCam network. AmeriFlux data are available, subject to a “fair use” policy, through an online portal (http://ameriflux.lbl.gov). The data generally include continuous (i.e. gap-filled) measurements of the net ecosystem exchange of CO_2_ (NEE), as well as latent and sensible heat fluxes, and environmental data (air temperature, humidity, wind speed and direction, solar radiation, soil temperature and soil water content, etc.), all at a 30-minute time step. Estimates of canopy photosynthesis and ecosystem respiration, derived from the data using an empirical model^59^, are also typically available. A set of scripts which will download Level 2 data from the AmeriFlux data archive is available here: https://github.com/khufkens/amerifluxr.

Nature’s Notebook, an effort of the USA National Phenology Network (USA-NPN), is a national citizen science program that engages both amateur and professional naturalists to make phenological observations of plants and animals using a standardized protocol^60^, and to contribute these to an online database. These data can be used to provide a direct, biological context for the PhenoCam time series and transition dates presented here. They could also be used for validation and testing of phenological models^24,61^ developed with, or calibrated to, PhenoCam data. Data from Nature’s Notebook are available for download through an online interface (https://www.usanpn.org/results/data).

Satellite remote sensing, e.g. from the Landsat (data available at http://landsat.usgs.gov/) or MODIS (MODerate Resolution Imaging Spectrometer; data available at http://modis.gsfc.nasa.gov/data/ or using an online interface at http://daac.ornl.gov/MODIS/) instruments, should be useful for placing data from individual PhenoCam sites in a broader (local-to-regional-to-global) context^20,21,25,27^. A perl client that will extract MODIS time series for a specified location (and for a window of pixels around that location) is available here:https://github.com/khufkens/modis-land-product-subset.

Finally, the original imagery from which the data presented here were derived is all freely available through the PhenoCam project web page (http://phenocam.sr.unh.edu/). The online interface permits users to browse the image archive by site, year, month, and day to quickly bring up the image associated with any data line in the all-image data files (i.e. Data Record 3). Users may also download imagery, on a site-by-site basis, for a specified date and time range. Colour channel information, for a user-defined ROI, can be extracted and processed to daily and three-day products, using the stand-alone precompiled PhenoCam GUI application (https://github.com/khufkens/phenocam-gui) or the PhenoPix R package (http://r-forge.r-project.org/projects/phenopix/)^62^.

The software routines used to generate the data sets presented here are publicly available for reuse by the community. Code for image processing, including extraction of colour information, and generation of “all-image”, and “summary” time series data files, is available at https://github.com/tmilliman/python-vegindex/, while an R package^57^for time series processing, including interpolation and uncertainty characterization, as well as outlier detection and transition date extraction, is available at https://khufkens.github.io/phenocamr/.

## Additional information

**How to cite this article:** Richardson, A. D. *et al.* Tracking vegetation phenology across diverse North American biomes using PhenoCam imagery. *Sci. Data* 5:180028 doi: 10.1038/sdata.2018.28 (2018).

**Publisher’s note:** Springer Nature remains neutral with regard to jurisdictional claims in published maps and institutional affiliations.

## Supplementary Material



## Figures and Tables

**Table 1:** Vegetation type abbreviations for ROIs (region of interests), and the corresponding number of site years of data included in PhenoCam dataset described here.

**Abbreviation**	**Description**	**Site years in Dataset**
AG	agriculture	50
DB	deciduous broadleaf	392
DN	deciduous needleleaf	4
EB	evergreen broadleaf	2
EN	evergreen needleleaf	80
GR	grassland	121
MX	mixed vegetation (generally EN/DN, DB/EN, or DB/EB)	5
SH	shrubs	46
TN	tundra (includes sedges, lichens, mosses, etc.)	22
WT	wetland	11
NV	non-vegetated	14
RF	reference panel	
XX	unspecified	
XX is used on the PhenoCam project page for preliminary ROIs that have not been fully vetted and quality-controlled (or that have been deemed of insufficient quality for general release), and as such no ROIs of type XX are included in the dataset described here.		

## Box

#

# ROI List for coweeta

#

# Site: coweeta

# Veg Type: DB

# ROI ID Number: 0001

# Owner: mtoomey

# Creation Date: 2012-09-05

# Creation Time: 11:42:00

# Update Date: 2012-09-05

# Update Time: 11:42:00

# Description: full canopy including deciduous and subdominant conifers

#

start_date,start_time,end_date,end_time,maskfile,sample_image

2011-04-14,00:00:00,2012-11-08,14:31:57,coweeta_DB_0001_01.tif,coweeta_2011_04_08_143030.jpg

2012-11-08,15:01:00,9999-12-31,23:59:59,coweeta_DB_0001_02.tif,coweeta_2012_11_09_113132.jpg

## Box

#

# ROI color statistics time series forcoweeta

# Site: coweeta

# Veg Type: DB

# ROI ID Number: 1

# Lat: 35.05959

# Lon: -83.42798

# Elev: 680

# UTC Offset: -5

# Resize Flag: False

# Creation Date: 2016-03-29

# Creation Time: 14:41:08

# Update Date: 2017-01-13

# Update Time: 04:19:18

#

date,local_std_time,doy,filename,solar_elev,exposure,mask_index,gcc,rcc,\

r_mean,r_std,r_5_qtl,r_10_qtl,r_25_qtl,r_50_qtl,r_75_qtl,r_90_qtl,r_95_qtl,\

g_mean,g_std,g_5_qtl,g_10_qtl,g_25_qtl,g_50_qtl,g_75_qtl,g_90_qtl,g_95_qtl,\

b_mean,b_std,b_5_qtl,b_10_qtl,b_25_qtl,b_50_qtl,b_75_qtl,b_90_qtl,b_95_qtl,\

r_g_correl,g_b_correl,b_r_correl

2011-04-14,06:01:32,104,coweeta_2011_04_14_060132.jpg,-0.51068,NA,1,0.33265,0.33957,48.46301,\

20.88536,13,22,35,48,61,74,83,47.47462,19.77350,15,23,35,47,59,72,81,46.78017,18.04348,\

18,25,35,46,57,69,78,0.97237,0.92784,0.89355

[continues...]

## Box

#

# 1-day summary product time series for coweeta

#

# Site: coweeta

# Veg Type: DB

# ROI ID Number: 0001

# Lat: 35.05959

# Lon: -83.42798

# Elev: 680

# UTC Offset: -5

# Image Count Threshold: 1

# Aggregation Period: 1

# Solar Elevation Min: 10.0

# Time of Day Min: 00:00:00

# Time of Day Max: 23:59:59

# ROI Brightness Min: 100

# ROI Brightness Max: 665

# Creation Date: 2017-02-20

# Creation Time: 09:59:45

# Update Date: 2017-03-06

# Update Time: 05:19:00

# Final Processing Date: 2017-03-06

# Final Processing Time: 20:18:10

#

date,year,doy,image_count,midday_filename,midday_r,midday_g,midday_b, midday_gcc,midday_rcc,\

r_mean,r_std,g_mean,g_std,b_mean,b_std,gcc_mean,gcc_std,gcc_50,gcc_75,gcc_90,\

rcc_mean,rcc_std,rcc_50,rcc_75,rcc_90,max_solar_elev,snow_flag,\

outlierflag_gcc_mean,outlierflag_gcc_50,outlierflag_gcc_75,outlierflag_gcc_90,\

smooth_gcc_mean,smooth_gcc_50,smooth_gcc_75,smooth_gcc_90,\

smooth_rcc_mean,smooth_rcc_50,smooth_rcc_75,smooth_rcc_90,\

smooth_ci_gcc_mean,smooth_ci_gcc_50,smooth_ci_gcc_75,smooth_ci_gcc_90,\

smooth_ci_rcc_mean,smooth_ci_rcc_50,smooth_ci_rcc_75,smooth_ci_rcc_90,int_flag

2011-0414,2011,104,20,coweeta_2011_04_14_120136.jpg,85.67173,75.03478,36.48126, 0.38052,0.43447,\

86.51166,9.91545,74.77882, 7.53882,41.37125,4.47597, 0.36936,0.03041,0.37652,0.37871,0.38066,\

0.42636,0.03012,0.42873,0.43566,0.4532,64.40871,2,0,0,0,0,0.3735,0.37768,0.38103,0.38318,\

0.4284,0.4302,0.43726,0.45111,0.00865,0.00934,0.00957,0.0096,0.0155,0.01616,0.01704,0.01864,NA

[continues...]

## Box

# An extract from this file is included below; for display purposes, the lines have been broken with a “\” character.

# Transition date estimate for coweeta

#

# Site: coweeta

# Veg Type: DB

# ROI ID Number: 0001

# Aggregation period: 1

# Year min: 2011

# Year max: 2015

# Final Processing Date: 2017-03-06

# Final Processing Time: 20:18:10

# Spline RMSE gcc_mean: 0.11807

# Spline RMSE gcc_50: 0.12551

# Spline RMSE gcc_75: 0.13474

# Spline RMSE gcc_90: 0.14585

#

sitename,veg_type,roi_id,direction,gcc_value,transition_10,transition_25,transition_50,\

transition_10_lower_ci,transition_25_lower_ci,transition_50_lower_ci,\

transition_10_upper_ci,transition_25_upper_ci,transition_50_upper_ci,\

threshold_10,threshold_25,threshold_50,min_gcc,max_gcc

coweeta,DB,0001,rising,gcc_90,2012-03-29,2012-04-12,2012-04-17,2012-03-25,2012-04-11,\

2012-04-16,2012-04-02,2012-04-14,2012-04-18,0.37823,0.39413,0.43275,0.36699,0.49223

[continues...]

## References

[d1] ORNL Distributed Active Archive Center Richardson A.D. *et al.* 2017 https://doi.org/10.3334/ORNLDAAC/1511

[d2] ORNL Distributed Active Archive Center Milliman T. *et al.* 2017 https://doi.org/10.3334/ORNLDAAC/1560

